# A fast and efficient method for preparation of high-quality RNA from fungal mycelia

**DOI:** 10.1186/1756-0500-6-71

**Published:** 2013-02-26

**Authors:** Ulrike Schumann, Neil A Smith, Ming-Bo Wang

**Affiliations:** 1 Commonwealth Scientific and Industrial Research Organisation, Plant Industry, Canberra, ACT 2601, Australia

**Keywords:** Fungi, RNA isolation, Fungal growth, Mycelium

## Abstract

**Background:**

Fungal RNA samples are usually isolated from fungal mycelia grown in liquid culture, which relies on prolific growth of the fungus in liquid media. The fungal biomass is then collected by vacuum filtration, which can result in low recovery for samples with reduced biomass due to poor growth in liquid media.

**Findings:**

Here we report an alternative culturing method, based on growth on solid media which is independent of the ability of a fungus to grow in liquid culture. We show that growth on solid media overlayed with a nylon membrane is superior to other culturing methods, producing large amounts of biomass and allowing for easy harvesting of fungal mycelia. Furthermore, we show that mycelium harvested with this method yielded high-quality RNA, superior to RNA isolated from liquid grown mycelium. We also show that inclusion of a second chloroform extraction step in the procedure significantly increases RNA yield.

**Conclusions:**

This method is particularly useful for fungal species that show poor or no growth in liquid media, but are easily cultured on solid media. Culturing can be performed on small petri dishes, which significantly reduces handling and therefore allowing growth and isolation of RNA from multiple strains in a high throughput manner. The obtained RNA samples are of high quality in sufficient quantities for several northern blot experiments or quantitative RT-PCR experiments.

## Findings

### Introduction

For isolation of nucleic acids from mycelia, the fungus is generally grown in liquid media [1] because fungal material can be difficult to harvest from standard solid media such as agar plates. Liquid cultures can be grown as a static culture, submerged in a large volume of media or aerial in a petri dish, or as a shaking culture. Static culturing methods often require filtration and washing of the fungal biomass to remove excess liquid and contaminating residues from the culture medium, whereas for shaking cultures the fungal material can be harvested by centrifugation [2,3]. If mycelial biomass is obtained from agar plates, it is essential to minimise the agar carryover when harvesting the material. RNA is then isolated using either phenol/SDS based methods [4], guanidinium thiocyanate [5,6] or commercially available RNA isolation kits [7,8]. However, many fungal strains grow poorly in liquid media, making it difficult and time-consuming to obtain sufficient material for RNA isolation. Furthermore, hyphal biomass can be difficult to pellet by centrifugation. We therefore developed an alternative method based on growth of the fungal material on solid medium overlayed with a nylon membrane, which proved to be fast and simple and yielded high quality RNA sufficient for multiple northern blot hybridization experiments.

We tested the method with two different fungal species, the necrotrophic fungus *Fusarium oxysporum* f.sp. *conglutinans* and the mycorrhizal fungus *Rhizoctonia solani*. For comparison, fungal biomass was also obtained by growth in liquid static and liquid shaking cultures and on solid agar without a nylon membrane overlay. RNA was isolated from the harvested fungal materials using TRIzol Reagent and the isolated RNA samples were compared for their quality and quantity.

### Methodology

#### Growth of fungi

**Figure 1:**
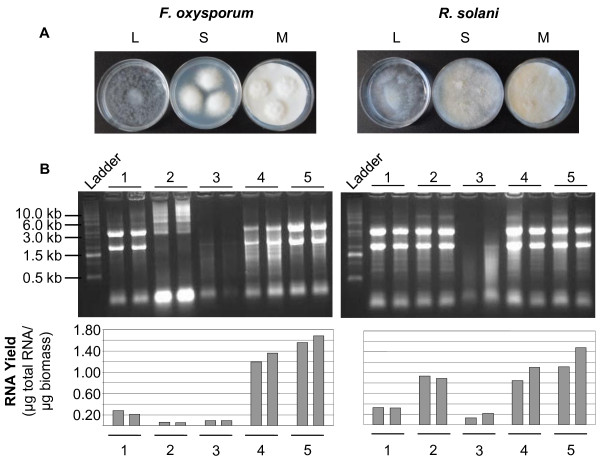
**(A) Growth of *Fusarium oxysporum *f.sp. *conglutinans *(left) and *Rhizoctonia solani *(right) 4 days after inoculation in static liquid culture (L), on solid media without membrane overlay (S) or solid media with nylon membrane overlay (M).** Growth of *F. oxysporum* in static liquid culture was poor and even after prolonged incubation a coherent mycelial mat was never formed. In contrast, growth of *R. solani* in static liquid culture was such that a coherent mycelia mat was obtained, which could easily be removed using a pair of tweezers. Growth of the replicate cultures was comparable and only one example is shown here. (**B**) Integrity (upper panels) and yield (lower panels) of total RNA samples isolated from *F. oxysporum* (left) and *R. solani (right)*. 5 μg of total RNA were separated on a 1.3% formaldehyde agarose gel. M – 0.5-10 kb RNA Ladder (Invitrogen, Mulgrave, VIC, Australia). 1 – static liquid culture, 2 – shaking liquid culture, 3 – solid media, 4 – solid media with membrane overlay, 5 – solid media with membrane overlay and two chloroform extractions.

Fungal biomass was obtained by inoculating the following media with 5 μl of a -80°C glycerol stock of *F. oxysporum* spores or with a small agar plug from a fresh plate of *R. solani*: i) 10 ml of liquid ^1^/_2_ strength Potato Dextrose Broth (PDB) (Sigma-Aldrich, Castle Hill, NSW, Australia) in a 50 mm petri dish kept static, ii) 25 ml of liquid ^1^/_2_ strength PDB in a 250 ml flask kept shaking at 200 rpm after inoculation, iii) 10 ml of ^1^/_2_ strength Potato Dextrose Agar (PDA) (Sigma-Aldrich, Castle Hill, NSW, Australia) in a 50 mm petri dish without nylon membrane overlay, iv) 10 ml of ^1^/_2_ strength PDA in 50 mm petri dish with nylon membrane overlay. All cultures were incubated at 28°C for 4 days (Figure 1A). Hybond nylon membranes were obtained from Amersham (GE Healthcare, Rydalmere, NSW, Australia). We have used Hybond-N, Hybond-N^+^ and Hybond-XL membranes (pore size of 0.45 μm) with equal results, however other types of membranes are likely to perform equally well. All growth methods were carried out in biological duplicates, with the membrane overlay culture further duplicated for analysis of differences between one and two chloroform extraction steps.

#### Harvest of fungal material

Growth of *F. oxysporum* in static liquid culture was poor (Figure 1A) and harvest of the mycelium by vacuum filtration through filter paper, the conventional method, was not possible since most of the mycelial mass could not be recovered. However, *R. solani* did form a coherent mycelial mat in static liquid culture that could easily be lifted with a pair of tweezers (Figure 1A). The biomass of all liquid cultures was collected by centrifugation at 16,000 × g for 5 minutes. Mycelia grown on solid media without nylon membrane overlay were harvested by scraping the surface using a clean scalpel, however it was difficult to dislodge the hyphae from the agar plates and consequently agar carryover occurred. Harvest of mycelial fractions from the nylon membrane proved to be the easiest. We have used nylon membranes in our experiments, however any type of membrane should work equally well to grow fungal material while providing a strong surface. The membrane can simply be lifted from the agar plate and the hyphae removed using a clean scalpel. The weight of all harvested material is listed in Table 1.

**Table 1:** **Purity of total RNA fractions isolated from *Fusarium oxysporum *f.sp. *conglutinans *and *Rhizoctonia solani***

	**Biomass (g)**	**Yield (μg RNA/mg biomass)**	^**260**^**/**_**280**_	^**260**^**/**_**230**_
			**ratio**	**ratio**
**Fusarium oxysporum**				
static liquid culture	0.23^a^	0.278	2.02	0.96
static liquid culture	0.17^a^	0.215	1.98	0.98
shaken liquid culture	0.20^a^	0.058	1.84	0.62
shaken liquid culture	0.20^a^	0.051	1.82	0.50
solid media no overlay	0.24^b^	0.094	1.86	0.26
solid media no overlay	0.51^b^	0.093	1.74	0.37
membrane overlay	0.12	1.192	1.93	1.50
membrane overlay	0.11	1.362	1.93	1.56
membrane overlay (2 chloroform)	0.12	1.556	2.02	1.61
membrane overlay (2 chloroform)	0.12	1.684	2.00	1.50
**Rhizoctonia solani**				
static liquid culture	0.38^a^	0.324	1.97	0.63
static liquid culture	0.37^a^	0.320	1.98	0.65
shaken liquid culture	0.28^a^	0.934	1.97	0.95
shaken liquid culture	0.35^a^	0.887	1.92	1.09
solid media no overlay	0.35^b^	0.129	1.60	0.18
solid media no overlay	0.16^b^	0.211	1.91	0.51
membrane overlay	0.20	0.845	1.83	0.59
membrane overlay	0.19	1.103	1.98	0.90
membrane overlay (2 chloroform)	0.19	1.110	2.00	0.71
membrane overlay (2 chloroform)	0.17	1.478	2.01	0.91

Measurements were performed using a Nanodrop reader.

^a^ Denotes samples where biomass measurements include residual water, all other samples are fresh weight. ^b^ Samples contained traces of agar, which resulted in gelatinous RNA pellets that were difficult to dissolve.

#### RNA isolation

The harvested fungal material was ground to a fine powder in liquid N_2_, then 2 ml TRIzol Reagent (Invitrogen, Mulgrave, VIC, Australia) was added and the sample ground further until the slurry had thawed. The sample was split into two equal volumes and transferred to 2 ml Eppendorf tubes. After 5 min incubation at room temperature (RT) 300 μl of chloroform was added, the tube shaken vigorously for 15 seconds and allowed to stand at RT for 3 minutes. The sample was then centrifuged at 13,000 × g for 15 minutes at 4°C. One nylon membrane-derived sample was subjected to a second chloroform extraction as above. The supernatant from the final extraction step was transferred to a clean 1.5 ml Eppendorf tube and the RNA precipitated with 500 μl isopropanol at −20°C for 2 hours or longer (over night when small RNAs are to be recovered). Precipitated RNA was collected by centrifugation at 13,000 × g for 15 minutes at 4°C, the pellet washed with 1 ml of ice cold 75% ethanol and air dried briefly at RT. The RNA pellets were resuspended in 20 μl of nuclease free water and the two duplicate tubes combined.

#### Assessment of RNA quantity and quality

RNA concentration and purity were measured using a NanoDrop Spectrophotometer (Biolab ND-1000) (Thermo Fisher Scientific, Scoresby, VIC, Australia) (Table 1). All chemicals and reagents were obtained from Sigma (Sigma-Aldrich, Castle Hill, NSW, Australia). To visually assess integrity and purity of the RNA, samples were analysed by formaldehyde agarose gel electrophoresis (1.3% agarose, 5% formaldehyde in 1 × MOPS buffer (20 mM 3-[N-morpholino]propane-sulfonic acid, 5 mM sodium acetate, 1 mM EDTA)). Five μg of total RNA was mixed with 3.5 μl formaldehyde solution (40% w/v), 10 μl formamide, 2 μl of 10 × MOPS buffer and 1 μl of ethidium bromide (1 mg/ml) in a total volume of 20 μl, then heated to 95°C for 5 min and 5 μl RNA loading dye (8% ficoll, 0.02% bromophenol blue, 0.04% xylene cyanol FF) added prior to loading. Integrity of the RNA fractions is shown in Figure 1B.

### Discussion

We found that fungal materials harvested from solid media without membrane overlay yielded gelatinous RNA pellets, which were hard to re-suspend, presumably due to contamination with agar. Consequently, the RNA is of poor quality showing clear degradation (Figure 1B). RNA of good quality and purity was obtained from both static liquid or membrane overlay cultures for both fungal species. However, total RNA yields from static cultures were considerably lower than from membrane overlay cultures (Table 1 and Figure 1B). The addition of a second chloroform step during isolation from membrane overlay cultures improved the RNA yield noticeably, presumably due to removal of contaminating polysaccharides that can reduce RNA solubility.

The method described here has several advantages over existing methods. It is fast, simple, very easy to follow and independent of the ability of the fungal species to grow in liquid medium. Using TRIzol Reagent to extract RNA abolishes the need to make up complicated buffers that are required for other methods. Furthermore, most fungal studies are carried out using mycelium, however many fungi preferentially produce asexual spores in liquid culture, which are morphologically different to mycelium. Therefore isolating RNA from mycelial fractions rather than spores is preferential. Growing fungal material on a nylon membrane greatly simplifies experimental set-up and handling. We suggest that other types of membranes such as cellophane membrane or other strong surfaces can also be used to overlay agar plates (as long as sufficient access to the nutrients is guaranteed), as these will provide a resilient surface for removal of the biomass. Using an overlay technique and subsequent removal of the biomass with a scalpel, removes the need for centrifugation and washing steps that are required when using liquid cultures, an advantage even for fungi that grow well in liquid culture. Indeed we obtained higher RNA yields from mycelium grown on a membrane compared to mycelium grown in liquid culture for *R. solani*, a fungus that grows reasonably well in liquid culture (Figure 1A), further emphasising that this method is superior to liquid growth methods. Furthermore, we obtained a 3–6 fold increased yield of RNA when compared with the static liquid culturing method. Although a second chloroform step increased RNA yield and quality, for researchers wishing to save time and obtain reasonable clean RNA, this step is not essential and the quality of the obtained RNA is sufficient for Northern blot analysis or RT-PCR experiments.

**Figure 2:**
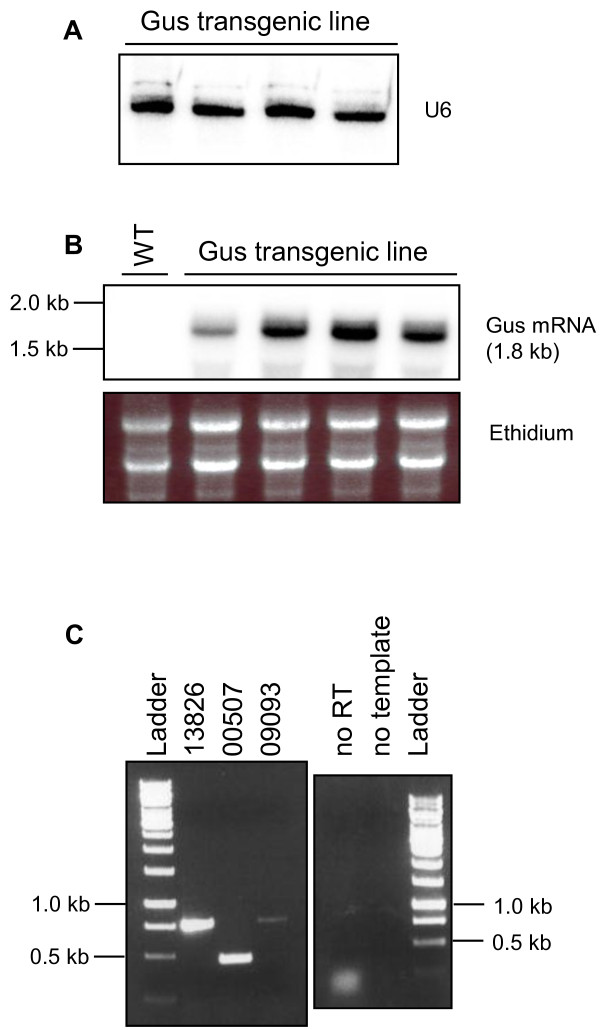
**Example of northern blot hybridisation detecting small RNA (A) and mRNA (B) fractions from wild-type (WT) and a transgenic *Gus *expressing *Fusarium oxysporum *lines.** For small RNA northern analysis 15 μg of total RNA were separated on a 17% polyacrylamide gel and probed for the U6 small nuclear RNA. For mRNA northern analysis, 10 μg of total RNA were hybridised with a probe detecting sense *Gus* mRNA. The ethidium staining of the rRNA is shown as loading control. (**C**) Total RNA from *Fusarium oxysporum* was used for reverse transcription and PCR to amplify parts of three independent *Fusarium* genes (FOXG_13826, FOXG_00507, FOXG_09093; Broad Institute *Fusarium* Comparative Database). Total RNA samples were DNase treated and reverse transcription and PCR carried out using the One-Step RT-PCR Kit (Qiagen, Chadstone, VIC, Australia) using gene specific primers (left panel). A control PCR to confirm that the RNA was free of DNA contamination was performed prior to the reverse transcription reaction (right panel).

We have routinely used this method to isolate total RNA (containing mRNA as well as small RNAs) from multiple *F. oxysporum* lines in parallel and routinely obtained ~100 μg of total RNA from mycelia grown for 4 days on 10 ml of solid media overlayed with a Hybond membrane, performing two chloroform steps. The isolated RNA was used in Northern blot hybridisation experiments (mRNA and small RNA detection), 5^′^ RACE and RT-PCR analyses. Figure 2 shows an example of northern blot hybridization utilising RNA isolated with this method. Although we have only performed RNA isolation from small amounts of fungal biomass, this method is reasonably easy to adapt to large-scale cultures following the manufacturer’s instructions of the TRIzol Reagent. In addition to RNA isolation, we have also successfully used the membrane overlay culturing technique for DNA and protein isolation. We expected that this method is suitable for all fungal species that can be cultured on solid media even if special culture media is required.

## Competing interests

The authors declare that they have no competing interests.

## Authors’ contributions

US designed the study, carried out experiments and wrote the manuscript. NAS carried out experiments and helped drafting the manuscript. MBW designed the study and wrote the manuscript. All authors read and approved the final version.
